# A Simple Method of Producing a Reticulated Surface on the Palatal Side of Dental Bas-Plates

**Published:** 1884-03

**Authors:** George Brunton

**Affiliations:** Leeds. Eng.


### A Simple Method of Producing a Reticulated Surface on the Palatal Side of Dental Base-Plates.

BY GEORGE BRUNTON, LEEDS. ENG.

Cut a piece of sand-paper a little shorter than the plate, place the sand side next the plate, and pass it through the flatting mills; remove the sand-paper, anneal the plate, take a fresh piece of sand-paper and apply as before, rolling in the opposite direction. For 20-carat gold this is enough, but for hard platina three or four rollings with sand-paper will be required to give it the desired roughness. Of course the plate must be a little thicker than the required strength to allow for the thinning out under the sand-paper. Dental plates treated in this manner are more easily struck, have a more artistic finish, are very much improved in the quality usually called suction, do not gather food, and are easily cleaned. Any grade of multicellular surface may be obtained by using coarse or fine sand-paper--do not use emery-paper, it will spoil the mills. The grade I prefer, and get the best results from, is produced by using Oakey's No. 3 sand-paper. The edges of palatal side of plate should be filed smooth before it is finally struck up.

